# Lactobacillus GG and other probiotics in pediatric food allergy treatment: a network meta-analysis

**DOI:** 10.3389/fnut.2025.1565436

**Published:** 2025-06-03

**Authors:** Li Xiaohua, Du Yiting, Li Qin, Zhai Yang, Wu Shumao, Peng Li, Pan Yi, Chen Lingmei, Liao Wenge, Li Maoxia

**Affiliations:** Chengdu Women’s and Children’s Central Hospital, School of Medicine, University of Electronic Science and Technology of China, Chengdu, China

**Keywords:** food allergy, pediatric, probiotics, network meta-analysis, SCORAD, Lactobacillus GG, quality of life

## Abstract

**Background:**

Food allergies are a significant health challenge in children, impacting quality of life and posing a burden on healthcare systems. Probiotics have been proposed as a potential treatment for food allergies, but their efficacy remains controversial. This systematic review and network meta-analysis aimed to assess the comparative effectiveness of different probiotics in managing pediatric food allergies.

**Methods:**

Following the Cochrane Handbook and PRISMA guidelines, a systematic search was conducted across PubMed, Cochrane Library, Web of Science, and Medline up to March 5, 2024. Randomized controlled trials (RCTs) evaluating probiotics for pediatric food allergies were included. The Cochrane risk of bias tool was used for quality assessment. Network meta-analyses were performed using random-effects models to calculate standardized mean differences (SMDs), odds ratios (ORs), and surface under the cumulative ranking curve (SUCRA) for different probiotics.

**Results:**

Sixteen RCTs involving 1,502 participants aged 1 month to 10 years were included. Eight probiotic interventions were analyzed. Lactobacillus GG (LGG) was identified as the most effective in reducing Scoring Atopic Dermatitis (SCORAD) scores (SMD = −4.24, 95% CI [−7.12, −1.36]; *p* < 0.05) and improving quality of life. For IgE regulation, *Lactobacillus acidophilus* (LB) demonstrated the greatest efficacy (*p* < 0.05). Publication bias was minimal for SCORAD and IgE outcomes, but some bias was detected for quality of life due to the limited number of studies.

**Conclusion:**

This study suggests that LGG is the most effective probiotic for improving clinical outcomes in pediatric food allergy management, particularly for SCORAD scores and quality of life. However, further high-quality RCTs are needed to validate these findings and explore the mechanisms underlying the differential efficacy of probiotic strains.

## 1 Introduction

Food allergies are a significant global health concern, particularly in children, with prevalence rates reaching 8% in children and 3% in adults (1–3). These conditions are characterized by adverse immune responses to dietary antigens and are associated with substantial healthcare burdens and reduced quality of life (2, 4). Patients and families alike live with lingering issues caused by allergic reactions to commonplace triggers like milk, peanuts, or eggs. Existing treatment approaches-da-dietary decrease and allergen-specific immunotherapies-do offer some help, yet they have limitations such as noncompliance, recurrent symptoms, and varied efficacy (5, 6). Such challenges underline the continued need for alternative and effective interventions.

Gut microbiota, which are a complex community of microorganisms in the gastrointestinal tract, are of critical importance in immune regulation, tolerance (7, 8). Early-life influences, such as cesarean delivery, formula feeding, or antibiotic use, can disrupt the gut composition and increase the risk for allergic diseases (9–12). Dysbiosis is a distinct form characterized by decreased microbial diversity and the demise of some beneficial bacteria, such as Bifidobacterium and Lactobacillus species; its involvement in food allergy initiation has been studied (12, 13). Additionally, microbial metabolites such as short-chain fatty acids (SCFAs), including butyrate, acetate, and propionate, help maintain immune tolerance by promoting the differentiation of Treg cells and that SCFAs enhanced intestinal barrier function (14, 15). Hence, the potential targeted therapies of the microbiota for the management of food allergies are highlighted.

Probiotics are defined as “live microorganisms which, when administered in adequate amounts, confer health benefits on the host (16–19),” and have become a potential treatment for allergies. Some such as *Lactobacillus rhamnosus* GG (LGG), and *Bifidobacterium bifidum* have demonstrated potential in alleviating food allergy symptoms through the restoration of gut microbial balance, enhancement of SCFA production, and cytokine synthesis, along with modulation of inflammatory responses (20, 21). Clinical studies have shown an overall reduction in SCORAD (Scoring Atopic Dermatitis) scores, improvement in health-related quality of life indices, and amelioration of immune markers like IgE levels (22–25). Despite probiotics being claimed for food allergy management, they have exhibited inconsistent efficacy. The discrepancies in results among studies can be attributed to differences in the probiotic strains, dosages, duration of treatment, and characteristics of participants. Such conflicting observations stress the need for further studies before reaching any conclusions. One aspect of the utility of probiotics, however, that is still not fully understood relates to the mechanism of their action. Proposed mechanisms include modulation of gut-associated lymphoid tissue (GALT) and cytokines. Apart from this, there are further limitations of the studies given that, most of the studies are with small sample sizes and the current random controlled trials (RCTs) mainly do not imply the necessary blinding and short follow-up periods to assess the long-term effectiveness and safety of probiotics. Further complicating the interpretation of results is the lack of consensus on the choice of strains, dosages, or duration of treatment. Particularly limited is long-term exposure of probiotics, raising questions about sustainable probiotic effects and the feasibility of risks for vulnerable groups like infants.

This study seeks to bridge these gaps by conducting a systematic review and network meta-analysis to assess the efficacy of probiotics in treating pediatric food allergy. This study aims to analyze important predefined outcomes, including SCORAD scores, IgE levels, and quality of life, with a goal of identifying the most effective probiotic strains and intervention protocols. Mechanisms of probiotic effects will be explored, and the development of a standardized, evidence-based guideline should be attained. The result of this research could help to improve clinical practice and push forward the field of microbiota-targeted therapies for food allergy.

## 2 Methods

### 2.1 Search strategy

This review follows guidelines and procedures as stated in the Cochrane Handbook for Systematic Reviews of Interventions and the PRISMA guideline for reporting systematic reviews and meta-analyses. The protocol was registered in PROSPERO with registration number CRD42024571197. Two independent researchers systematically searched databases including PubMed, Cochrane Library, Web of Science, and Medline for all randomized controlled trials (RCTs) meeting the inclusion criteria published up to March 5, 2024. Additionally, we reviewed the reference lists of prior systematic reviews and meta-analyses in the field to identify any missing literature.

### 2.2 Inclusion and exclusion criteria

All RCTs evaluating the efficacy of probiotics in the treatment of pediatric food allergies were included based on the following criteria: (1) Participants aged ≤18 years, diagnosed with food allergies based on established diagnostic criteria, with a chronic or relapsing history. (2) Probiotics administered orally, with clear specification of probiotic species, dose, and timing of administration. (3) Control groups receiving a placebo intervention. (4) Primary endpoints assessed at follow-up after the intervention period. (5) IgE levels, SCORAD scores, or quality of life indicators reported as outcome measures. (6) Studies providing sufficient data for analysis that met the predefined search criteria.

Studies were excluded for the following reasons: (1) Non-RCT designs, including observational studies, case reports, single-arm studies, reviews, meta-analyses, letters, protocols, and other non-original research articles. (2) Duplicate studies or studies unrelated to the research topic. (3) Publications in languages other than English. (4) Articles with only abstracts or those lacking access to full texts or sufficient outcome data. (5) Studies involving adult participants, animal models, or *in vitro*/*in vivo* experiments.

### 2.3 Literature screening

Two independent researchers systematically searched databases including PubMed, Cochrane Library, Web of Science, and Medline for all RCTs meeting the inclusion criteria published up to March 5, 2024. They reviewed the literature references from prior systematic reviews and meta-analyses in the field to identify any missing literature.

### 2.4 Data extraction

Two researchers independently retrieved literature using the search strategy and used NoteExpress software for deduplication. They initially screened titles and abstracts and then reviewed the full texts based on the inclusion and exclusion criteria to finalize the selected studies. Data were extracted into a pre-designed Excel sheet, including basic information (first author, publication year, age), sample size, interventions for experimental and control groups, and outcome measures. Disagreements were resolved by a third researcher.

### 2.5 Quality assessment

Two researchers independently assessed the quality of included studies using the Cochrane Risk of Bias Tool, which evaluates seven aspects: random sequence generation, allocation concealment, blinding of participants and researchers, blinding of outcome assessors, completeness of outcome data, selective reporting, and other biases. Each item was rated as high risk, low risk, or unclear risk. Any discrepancies were resolved with the help of a third researcher.

### 2.6 Statistical analysis

For outcome measures in the network meta-analysis, binary variables were analyzed using odds ratios (ORs), and continuous variables were analyzed using standardized mean differences (SMDs). Effect sizes and 95% confidence intervals (CIs) were calculated. Network meta-analysis was performed using a frequentist framework in Stata 18 software, utilizing the “network” and “mvmeta” packages. Surface under the cumulative ranking curve (SUCRA) values were calculated to compare the efficacy of different interventions. Evidence networks, comparison-adjusted funnel plots, and cumulative probability plots were generated to evaluate publication bias and the effectiveness of different interventions. Higher SUCRA values indicate better intervention effects.

Pairwise meta-analyses were conducted for interventions directly compared in at least two studies, while indirect comparisons were calculated using a network meta-analysis framework. These “back-calculated” estimates utilize shared comparators to infer the efficacy between treatments that were not directly compared. All analyses reported both direct, indirect, and pooled effect sizes with corresponding 95% CIs and *p*-values. Heterogeneity across studies was quantified using the I^2^ statistic, which describes the percentage of variation due to heterogeneity rather than chance. A value of I^2^ > 50% was considered moderate to high heterogeneity. SUCRA plots were generated to visualize the probability of each treatment being ranked best. This method is based on cumulative probability and should not be interpreted as analogous to ROC AUC curves. It instead reflects comparative rankings under a Bayesian/frequentist synthesis framework.

## 3 Results

### 3.1 Search results

**Figure 1:**
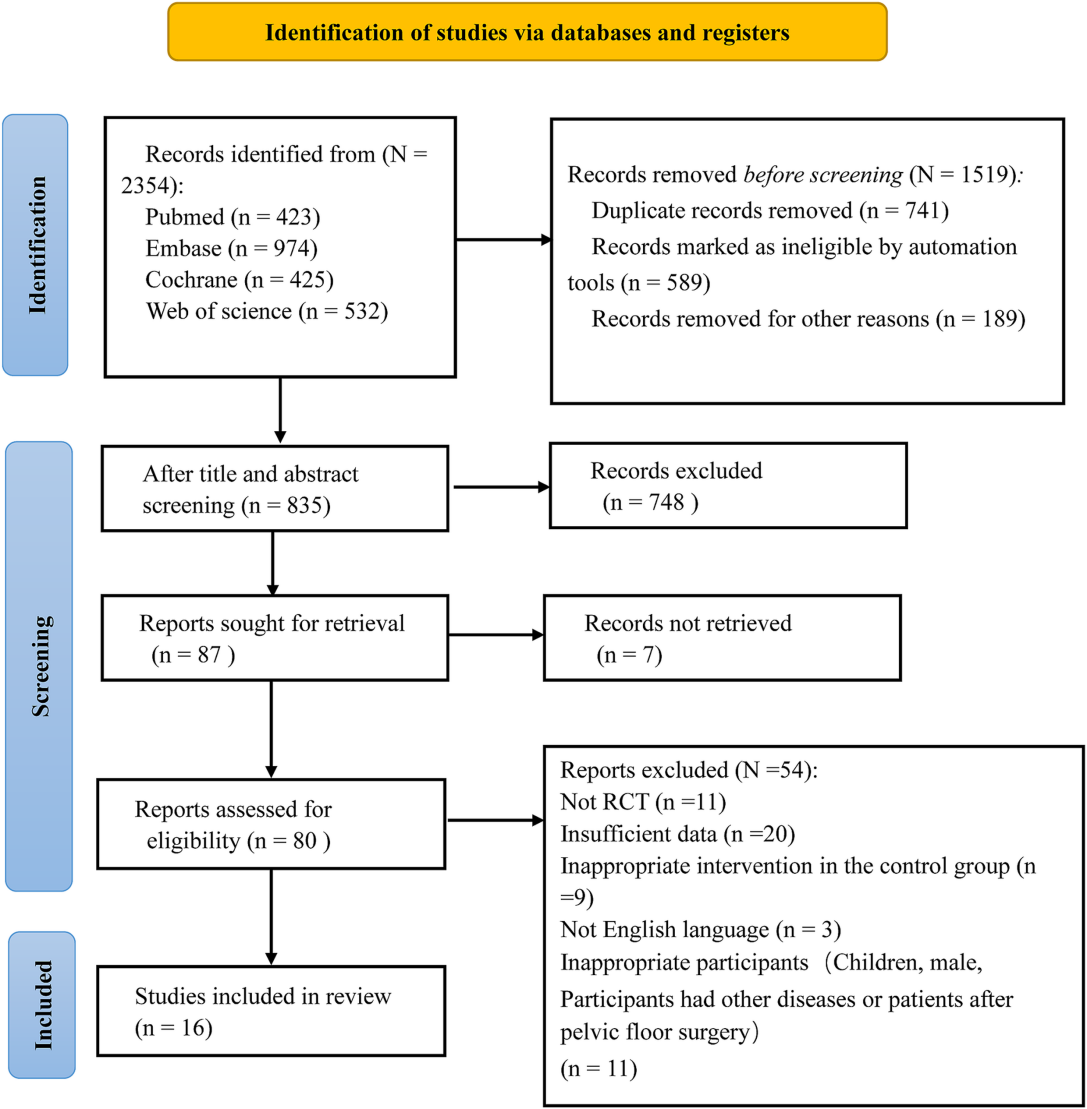
Artiele sereening proeess.

A total of 2,354 studies were initially retrieved. Among these, 741 were duplicates, a total of 589 studies marked as unqualified by the automated tool and 189 studies deleted for other reasons were excluded, and 748 irrelevant studies were excluded after reviewing the titles and abstracts. Additionally, 54 studies were excluded for not meeting the inclusion criteria (detailed exclusion reasons are shown in Figure 1). Finally, 16 eligible studies were included based on the selection criteria after a thorough review of the full texts (23, 26–40). The selection process is illustrated in Figure 1.

### 3.2 Characteristics of the included studies

The basic characteristics of the included studies are presented in Supplementary Table 1. A total of 16 RCTs with 1,502 participants were included, comprising 801 individuals in the intervention groups and 701 in the control groups (Table 1). The participants’ ages ranged from 1 month to 10 years. The studies were published between 1997 and 2022 and included eight probiotic interventions:

1. *Lactobacillus acidophilus* LB;
2. *Lactobacillus GG*; *Lactobacillus rhamnosus* (NP-Lrh);
3. A mixture of *Lactobacillus rhamnosus ŁOCK 0900*, *Lactobacillus rhamnosus ŁOCK 0908*, and *Lactobacillus casei ŁOCK 0918*;
4. *Lactobacillus paracasei* CNCM I-2116;
5. *Bifidobacterium lactis* CNCM I-3446;
6. A mixture of *Lactobacillus casei CRL431* and *Bifidobacterium bifidum* TMC3115;
7. *Lactobacillus acidophilus* L-92;
8. And a mixed probiotics group (*LGG*, *L. rhamnosus* LC705, *Bifidobacterium breve* Bbi99, and *Propionibacterium freudenreichii* ssp.).

**Table 1:** Evaluation of articles included in meta-analysis.

Author	Type of food allergy	Age T	Age C	NT	NC	Treatment T	Treatment C	Outcome	Blinding method	Randomization method	Article title
van der Aa (26)	Milk Allergy	5.0 (1.4)m	4.8 (1.5)m	36	39	Bifidobacterium M-16 V and Galactose/Fructooligosaccharide Mixture	Placebo	Questionnaire to assess the prevalence of respiratory symptoms and asthma medication use, measuring total IgE and specific IgE to airborne allergens	Double-blind	Computer randomization	Synbiotics prevent asthma-like symptoms in infants with atopic dermatitis
Atta (40)	Food Allergy	9.45 ± 3.70/9.45 ± 3.70y	7.91 ± 2.80/9.45 ± 3.70y	22/22	22/22	Lactobacillus LB Strain 10 Billion	Placebo	Asthma severity grading, total IgE measurement, and the Pediatric Asthma Quality of Life Questionnaire (PAQLQ)		Blind selected colored cards randomized into four groups	The effect of food elimination and probiotic supplementation in asthmatic children with food allergy
Basturk (27)	Milk Protein Allergy	68.75d	68.75d	48	52	Lactobacillus GG (LGG)	Placebo	Symptoms such as diarrhea, vomiting, mucus or blood in stools, abdominal pain or bloating, constipation, dermatitis, and irritability	Double-blind	Randomization	Investigation of the Efficacy of *Lactobacillus rhamnosus* GG in Infants With Cow’s Milk Protein Allergy: a Randomised Double-Blind Placebo-Controlled Trial
Brouwer (28)	Milk Allergy	3.9/3.8 m	3.6 m	17/16	17	Rhamnosus Lactobacillus (NP-Lrh)/Lactobacillus GG (NP-LGG)	Placebo	Atopic dermatitis (SCORAD) evaluation, IgE and a panel of food-specific IgE, and skin prick tests to evaluate milk allergy sensitization.Polyfunctional stimulation of peripheral blood mononuclear cells producing IL-4, IL-5, and IFN-g; inflammatory parameters include blood eosinophils, urinary eosinophil protein X, and fecal a-1 anti-trypsin	Double-blind	Randomization	No effects of probiotics on atopic dermatitis in infancy: a randomized placebo-controlled trial
Berni Canani (29)	Milk Allergy	5.0 (3.0 to 8.0)	5.0 (3.0 to 8.0)	98	95	Lactobacillus GG (LGG)	Placebo	Primary outcome is the occurrence of at least one case of AM (eczema, urticaria, asthma, and allergic rhinitis).Secondary outcome is the acquisition of tolerance	Double-blind	Randomization	Extensively hydrolyzed casein formula containing *Lactobacillus rhamnosus* GG reduces the occurrence of other allergic manifestations in children with cow’s milk allergy: 3-year randomized controlled trial
Cukrowska (23)	Milk Allergy	8.2 ± 6.1(4–23)	8.2 ± 6.1(4–23)	48	g	Rhamnosus Lactobacillus ŁOCK 0900, ŁOCK 0908, and Cheddar Lactobacillus ŁOCK 0918	Placebo	AD severity changes assessed by SCORAD index, total IgE, and allergen-specific IgE levels	Double-blind	Randomization	The Effectiveness of Probiotic *Lactobacillus rhamnosus* and *Lactobacillus casei* Strains in Children with Atopic Dermatitis and Cow’s Milk Protein Allergy: A Multicenter, Randomized, Double Blind, Placebo Controlled Study
Gore (30)	Milk Allergy	1.5 [18–25]/23 [10–25.5w]	23 [19–26]w	45/45	47	Subsidiary Cheddar Lactobacillus or Lactobacillus	Placebo	Eczema severity at 3 months (Atopic Dermatitis score, SCORAD).Secondary: SCORAD (other visits); Infant dermatitis quality of life (IDQoL); gastrointestinal permeability; urinary eosinophilic protein X; allergen-sensitization; allergic symptoms	Double-blind	Randomization	Treatment and secondary prevention effects of the probiotics *Lactobacillus paracasei* or *Bifidobacterium lactis* on early infant eczema:randomized controlled trial with follow-up until age 3 years
Hol (31)	Milk Allergy	4.3 (1.2 m)	4.1 (1.5)m	51	55	Cheddar Lactobacillus CRL431 and Lactobacillus Bifidobacterium Bb-12	Placebo	Clinical tolerance SCORAD score for CM at 6 and 12 months (hospitalization, wheezing, medication use, hormones, SPT positive reactions); T lymphocyte and B lymphocyte subgroups (CD31, CD31CD41, CD31CD81, and CD201) in peripheral blood were used, and viable bacterial strains were detected in fecal samples.	Double-blind	Computer randomization	The acquisition of tolerance toward cow’s milk through probiotic supplementation: A randomized, controlled trial
Isolauri (32)	Food allergies (Egg, Milk, and Wheat)	4.6 m	4.6 m	9	9	Lactic Acid Bacteria Bifidobacterium Bb-12 or Lactobacillus GG (ATCC 53103)	Placebo	Atopic eczema severity and intensity SCORAD score, macrophage colony-stimulating factor (GM-CSF), soluble intercellular adhesion molecule 1 (sICAM-1), and tumor necrosis factor-alpha (TNFa) to assess inflammation status, with the selection of soluble CD4 (sCD4), soluble CD8 (sCD8), and IL-2 soluble receptor alpha (IL-2 sRa).	Double-blind	Randomization	Probiotics in the management of atopic eczema
Jing (33)	Milk Protein Allergy	6. 38 ± 5. 52 m	6. 24 ± 5. 71 m	128	128	Bifidobacterium	Placebo	Allergy symptom scoring (gastrointestinal, respiratory, skin, and systemic allergy reflections); inflammatory factors (TNFα/IL-1β/IL-6/IL-10); antibodies (IgE/IgG2); efficacy in eczema reduction.	Double-blind	Computerized table method	*Bifidobacterium bifidum* TMC3115 ameliorates milk protein allergy in by affecting gut microbiota: A randomized double-blind control trial
Loke (34)	Peanut Allergy	1–5/6-10y	1–5/6-10y	79	83	Rhamnosus Lactobacillus ATCC 53103, 2 × 10^1^⁰ (Probiotics and Peanut Oral Immunotherapy)	Peanut oral immunotherapy	After-treatment peanut skin prick test wheel size at 8 weeks and 12 months, peanut-specific and peanut component-specific IgE and IgG4 (Ara h 1, h 2, and h 3), as well as their changes from baseline. At 12 months after treatment completion, HRQOL was assessed via FAQLQ-PF, and the incidence of abdominal pain, vomiting, and systemic organ-related respiratory diseases (including preferred terms allergic cough, allergic respiratory tract, and respiratory adverse events) was significantly reduced.	Double-blind	Computer-generated block randomization	Probiotic peanut oral immunotherapy versus oral immunotherapy and placebo in children with peanut allergy in Australia (PPOIT-003): a multicentre, randomised, phase 2b trial
Majamaa (35)	Milk Allergy	0.6–8.5(4.4)m	0.6–8.5(4.4)m	13	14	Lactobacillus GG (5 × 10⁵ CFU/gin)	Placebo	SCORAD method rating, serum ECP measurement, fecal l-antitrypsin, tumor necrosis factor-, and eosinophilic cationic protein concentrations as markers of intestinal inflammation.	Double-blind	Randomization	Probiotics: A novel approach in the management of food allergy
Nakata (36)	Food Allergy	1.7 (0.9–3.0)	1.7 (0.9–3.0)	25	20	Lactobacillus L-92 (L-92)	Placebo	Atopic Dermatitis (SCORAD) index evaluation, Atopic Dermatitis score; white blood cells; TARC, thymus and activation-regulating chemotactic factor; lactate dehydrogenase; AST, aspartate aminotransferase; ALT, alanine aminotransferase.	Double-blind	Randomization	Additive effect of *Lactobacillus acidophilus* L-92 on children with atopic dermatitis concomitant with food allergy
Kirjavainen (37)	Milk Allergy	5.5 m	5.5 m	14/13	8	Live LGG Group/Heat-Inactivated LGG Group	Placebo	Atopic dermatitis (SCORAD) evaluation, intestinal microbiota	Double-blind	Not provided	Probiotic Bacteria in the Management of Atopic Disease: Underscoring the Importance of Viability
Tang (38)	Peanut Allergy	6.1 (2.4), 31y	6.1 (2.4), 31y	31	31	Rhamnosus Lactobacillus CGMCC 1.3724 (NCC4007)	Placebo	Tolerance, serum peanut-specific IgE (sIgE) and peanut-specific IgG4	Double-blind	Randomization	Administration of a probiotic with peanut oral immunotherapy: A randomized trial
Viljanen (39)	Milk Allergy	5.9 (1.8–10.8)/5.9 (1.8–10.8)m	5.9 (1.8–10.8)m	44/44	32	Rhamnosus Lactobacillus GG/LGG 5·10⁹ CFU, Rhamnosus L. LC705 (LC705) 5·10⁹ CFU, Bifidobacterium Bbi99 2·10⁸ CFU, and *Propionibacterium freudenreichii*	Placebo	Atopic Dermatitis Severity Score (SCORAD); Serum, CM, and Wheat-Specific IgE Concentrations Detected Using the Pharmacia CAP System RAST FEIA (Pharmacia Ltd., Uppsala, Sweden).	Double-blind	Computer-generated block randomization	Probiotics in the treatment of atopic eczema/dermatitis syndrome in infants: a double-blind placebo-controlled trial

### 3.3 Quality assessment of the selected studies

**Figure 2:**
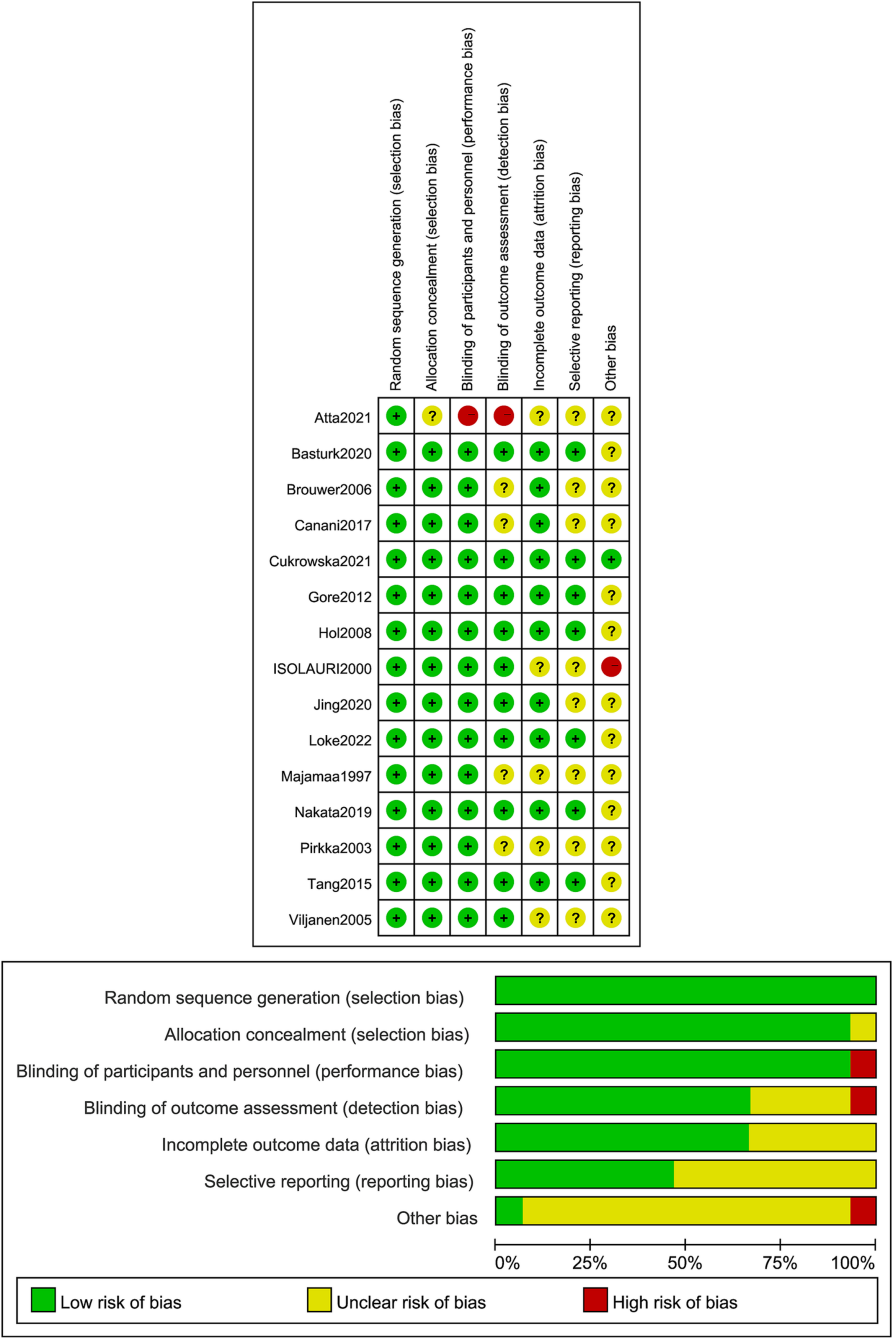
Proportion of projects with a risk of bias in the included literature.

Regarding random sequence generation, one study used random selection with colored cards, four used computer-based randomization, and one used random selection with colored cards, all assessed as low risk. Eight studies mentioned randomization but did not describe the specific methods used, and one study did not describe blinding, both rated as high risk. For blinding, 14 studies used double-blinding and were assessed as low risk, while one study did not mention blinding and was rated as high risk. Seven studies reported allocation concealment and blinding of outcome assessors, rated as low risk, while others were rated as unclear risk. Outcome data were complete in all studies, and no selective reporting of results was found, all rated as low risk. Other biases were rated as unclear risk (Figure 2). Regarding placebo formulations, most studies administered probiotics and placebos in capsule form; however, only a portion of the studies explicitly stated the formulation details.

### 3.4 Network Meta-analysis of SCORAD scores

**Figure 3:**
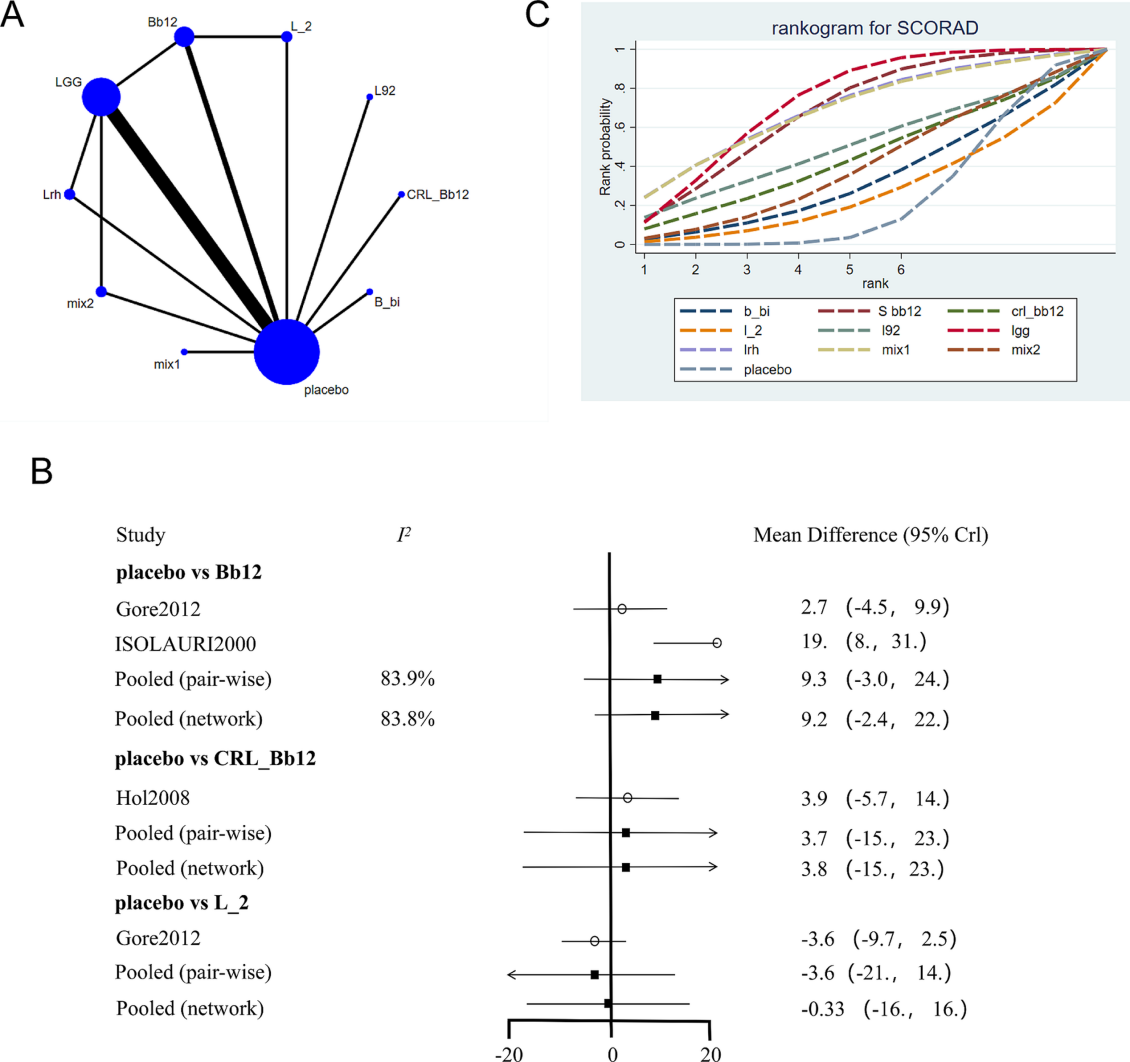
Network meta-analysis of SCORAD scores. **(A)** Network plot of interventions. Dot size reflects the total number of patients for each intervention, while line thickness indicates the number of direct comparisons between two interventions; longer and thicker lines denote stronger evidence bases. **(B)** Pairwise and network meta-analysis summary. The I^2^ statistic quantifies heterogeneity, with higher values indicating greater inconsistency. The heterogeneity *p* value was 0.086. All *p*-values for effect sizes are provided. **(C)** Cumulative ranking plot based on Surface Under the Cumulative Ranking (SUCRA) curves. A higher SUCRA value indicates a higher probability of being the best treatment. This ranking method is not equivalent to ROC/AUC, but reflects comparative efficacy across all treatments based on the cumulative probability distribution of ranks. Abbreviations: *LGG, Lactobacillus rhamnosus GG; LB, Lactobacillus acidophilus LB; NP-Lrh, Lactobacillus rhamnosus; CNCM, Lactobacillus paracasei CNCM I-2116; CRL431, Lactobacillus casei CRL431; TMC3115, Bifidobacterium bifidum TMC3115*; etc.

**Figure 4:**
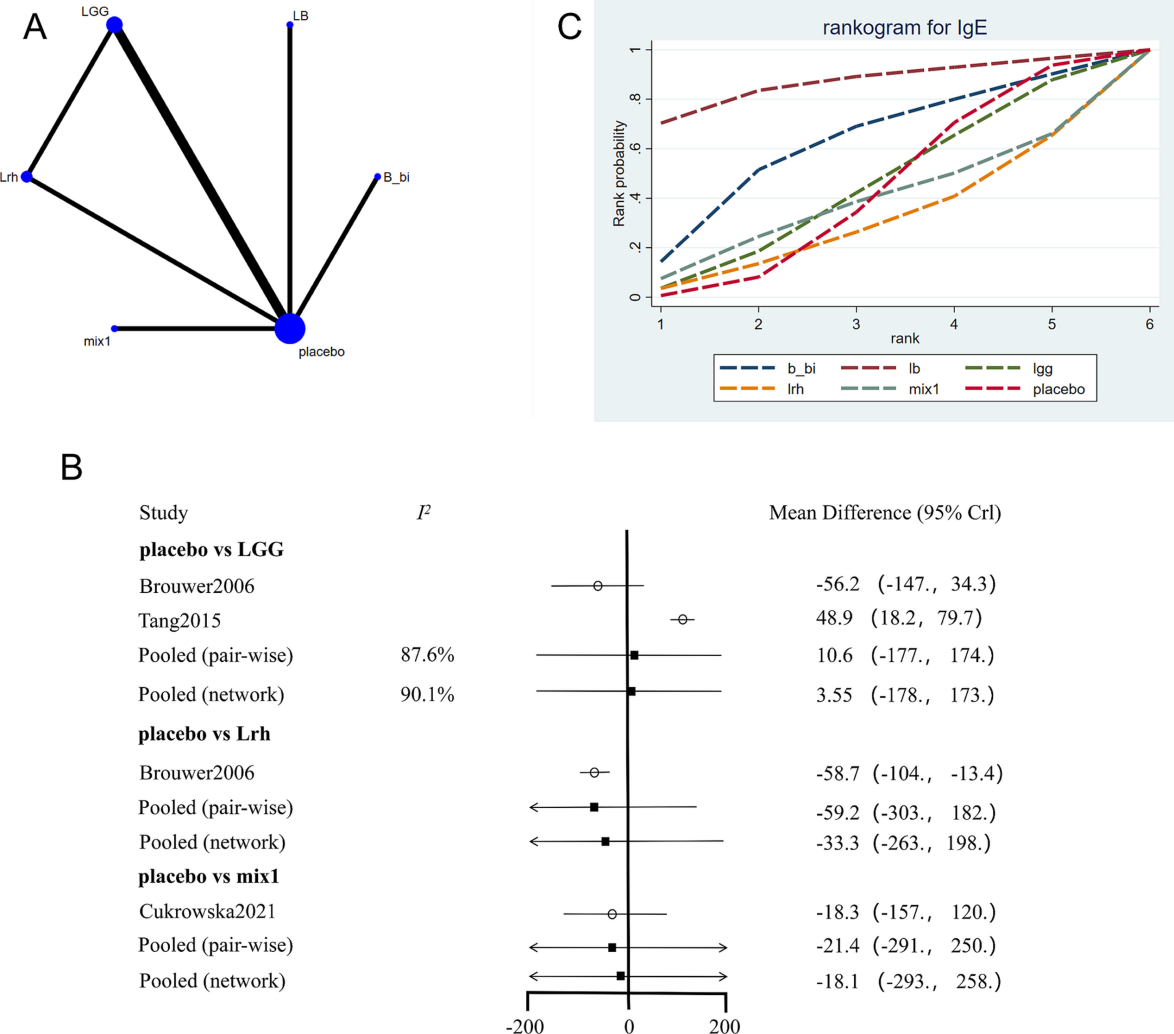
Network meta-analysis of IgE scores. **(A)** Network plot of interventions. Dot size reflects the total number of patients for each intervention, while line thickness indicates the number of direct comparisons between two interventions; longer and thicker lines denote stronger evidence bases. **(B)** Pairwise and network meta-analysis summary. The I^2^ statistic quantifies heterogeneity, with higher values indicating greater inconsistency. The heterogeneity p value was 0.547. All p-values for effect sizes are provided. **(C)** Cumulative ranking plot based on SUCRA curves. A higher SUCRA value indicates a higher probability of being the most effective treatment. Abbreviations: see Figure 3.

**Figure 5:**
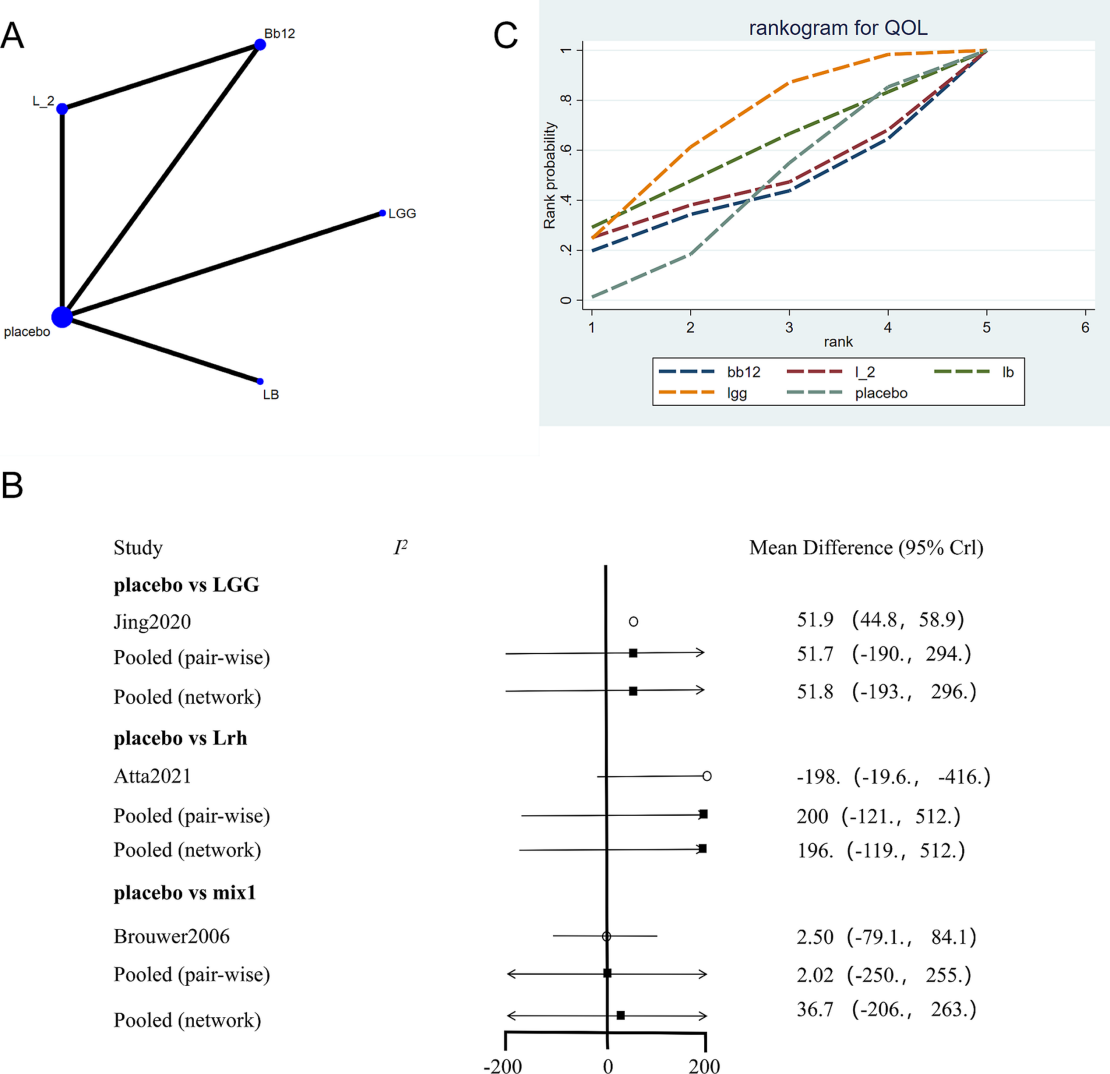
Network meta-analysis of quality of life scores. **(A)** Network plot of interventions. Dot size reflects the total number of patients for each intervention, while line thickness indicates the number of direct comparisons between two interventions; longer and thicker lines denote stronger evidence bases. **(B)** Pairwise and network meta-analysis summary. The I^2^ statistic quantifies heterogeneity, with higher values indicating greater inconsistency. The heterogeneity p value was 0.776. All p-values for effect sizes are provided. **(C)** SUCRA-based ranking curve for overall treatment efficacy. A higher SUCRA indicates better ranking probability. Abbreviations: see Figure 3.

The network evidence plot for SCORAD scores under different interventions was analyzed (Figure 3A). Placebo was the most commonly studied intervention. Data from different interventions were pooled, revealing high heterogeneity (Figure 3B). Random-effects models were used for analyzing the effects of various interventions. The results showed that placebo outperformed other interventions (*p* < 0.05). To further explore the efficacy of probiotics, a network meta-analysis was conducted. The results indicated that among the nine probiotic interventions, *Lactobacillus GG* (LGG) was the most effective in improving SCORAD scores (Figure 3C; Table 2). In panels C of Figures 3–5, the cumulative ranking plots illustrate the probability of each intervention being among the most effective. A higher area under the SUCRA curve (closer to 100%) suggests greater comparative efficacy. Unlike ROC curves, SUCRA plots rank probabilities rather than classify outcomes. For example, in Figure 3C, LGG shows the highest SUCRA value for SCORAD improvement, indicating it has the highest chance of being the most beneficial probiotic for this outcome. This pattern was consistent across the other two outcomes (IgE and QoL), strengthening the inference of LGG’s overall superiority.

**Table 2:** Network meta-analysis of SCORAD scores.

	B_bi	Bb12	CRL_Bb12	L_2	L92	LGG	Lrh	mix1	mix2	placebo
B_bi	B_bi	−7.98 (−29.36, 12.6)	−2.59 (−28.26, 23.02)	1.66 (−22.17, 24.98)	−4.2 (−31.24, 23.06)	−9.14 (−27.35, 10.62)	−9.01 (−34.02, 14.66)	−8.93 (−33.63, 15.97)	−1.81 (−24.9, 21.86)	1.32 (−15.58, 18.39)
Bb12	7.98 (−12.6, 29.36)	Bb12	5.45 (−16.96, 28.67)	9.62 (−6.72, 26.22)	3.87 (−20.14, 28.83)	−1.04 (−13.49, 13.55)	−1.03 (−22.18, 19.81)	−0.93 (−22.38, 21.32)	6.2 (−12.78, 26.54)	9.33 (−2.5, 21.99)
CRL_Bb12	2.59 (−23.02, 28.26)	−5.45 (−28.67, 16.96)	CRL_Bb12	4.22 (−21.39, 29.27)	−1.53 (−30.2, 27.22)	−6.43 (−26.91, 15.15)	−6.48 (−32.81, 18.98)	−6.35 (−32.86, 20.01)	0.78 (−23.9, 26.01)	3.9 (−15.37, 23.18)
L_2	−1.66 (−24.98, 22.17)	−9.62 (−26.22, 6.72)	−4.22 (−29.27, 21.39)	L_2	−5.78 (−32.2, 21.24)	−10.71 (−27.72, 8.2)	−10.68 (−34.87, 12.75)	−10.59 (−34.83, 14.26)	−3.46 (−25.54, 19.62)	−0.31 (−16.53, 16.42)
L92	4.2 (−23.06, 31.24)	−3.87 (−28.83, 20.14)	1.53 (−27.22, 30.2)	5.78 (−21.24, 32.2)	L92	−4.81 (−27.24, 18.51)	−4.91 (−32.97, 22.01)	−4.76 (−32.62, 23.01)	2.36 (−23.92, 29.16)	5.48 (−15.76, 26.74)
LGG	9.14 (−10.62, 27.35)	1.04 (−13.55, 13.49)	6.43 (−15.15, 26.91)	10.71 (−8.2, 27.72)	4.81 (−18.51, 27.24)	LGG	−0.05 (−19.19, 16.93)	0.1 (−20.47, 19.44)	7.28 (−9.17, 22.86)	10.37 (1.32, 18.18)
Lrh	9.01 (−14.66, 34.02)	1.03 (−19.81, 22.18)	6.48 (−18.98, 32.81)	10.68 (−12.75, 34.87)	4.91 (−22.01, 32.97)	0.05 (−16.93, 19.19)	Lrh	0.09 (−24.24, 25.88)	7.28 (−15.01, 31.01)	10.4 (−6.43, 28.33)
mix1	8.93 (−15.97, 33.63)	0.93 (−21.32, 22.38)	6.35 (−20.01, 32.86)	10.59 (−14.26, 34.83)	4.76 (−23.01, 32.62)	−0.1 (−19.44, 20.47)	−0.09 (−25.88, 24.24)	mix1	7.14 (−16.76, 31.46)	10.28 (−7.95, 28.33)
mix2	1.81 (−21.86, 24.9)	−6.2 (−26.54, 12.78)	−0.78 (−26.01, 23.9)	3.46 (−19.62, 25.54)	−2.36 (−29.16, 23.92)	−7.28 (−22.86, 9.17)	−7.28 (−31.01, 15.01)	−7.14 (−31.46, 16.76)	mix2	3.15 (−13.01, 18.85)
placebo	−1.32 (−18.39, 15.58)	−9.33 (−21.99, 2.5)	−3.9 (−23.18, 15.37)	0.31 (−16.42, 16.53)	−5.48 (−26.74, 15.76)	‘-10.37 (−18.18, -1.32)	−10.4 (−28.33, 6.43)	−10.28 (−28.33, 7.95)	−3.15 (−18.85, 13.01)	placebo

### 3.5 Network meta-analysis of IgE levels

The network evidence plot for IgE levels across different interventions was constructed (Figure 4A). Placebo was again the most commonly studied intervention. Data from different interventions showed high heterogeneity (Figure 4B), and random-effects models were applied. Results indicated that placebo had better effects compared to *Lactobacillus rhamnosus* (Lrh) but was less effective compared to LGG. A network meta-analysis further revealed that *Lactobacillus acidophilus* LB was the most effective intervention for reducing IgE levels (Figure 4C; Table 3).

**Table 3:** Network meta-analysis of IgE Levels.

	B_bi	LB	LGG	Lrh	mix1	placebo
B_bi	B_bi	−146.63 (−549.14, 254.23)	48.61 (−248.39, 358.48)	85.44 (−252.74, 422.81)	70.5 (−299.42, 439.79)	52.1 (−195.18, 297.05)
LB	146.63 (−254.23, 549.14)	LB	197.85 (−162.93, 567.34)	232.21 (−163.21, 627.9)	218.43 (−205.13, 639.48)	198.82 (−122.21, 521.08)
LGG	−48.61 (−358.48, 248.39)	−197.85 (−567.34, 162.93)	LGG	36.34 (−207.12, 264.31)	20.42 (−312.87, 345.28)	2.92 (−179.69, 173.95)
Lrh	−85.44 (−422.81, 252.74)	−232.21 (−627.9, 163.21)	−36.34 (−264.31, 207.12)	Lrh	−14.68 (−373.53, 346.12)	−33.45 (−265.79, 201.43)
mix1	−70.5 (−439.79, 299.42)	−218.43 (−639.48, 205.13)	−20.42 (−345.28, 312.87)	14.68 (−346.12, 373.53)	mix1	−18.4 (−293.73, 257.23)
placebo	−52.1 (−297.05, 195.18)	−198.82 (−521.08, 122.21)	−2.92 (−173.95, 179.69)	33.45 (−201.43, 265.79)	18.4 (−257.23, 293.73)	placebo

### 3.6 Network meta-analysis of quality of life

The network evidence plot for quality-of-life measures across different interventions was analyzed (Figure 5A). Placebo was the most commonly studied intervention. Data showed high heterogeneity, and a fixed-effects model was applied (Figure 5B). The results indicated that placebo outperformed other interventions (*p* < 0.05). However, in the network meta-analysis, LGG emerged as the most effective intervention for improving quality-of-life outcomes (Figure 5C; Table 4).

**Table 4:** Network meta-analysis of quality of life.

	B_bi	BbM_16	LB	LGG	Lrh	mix1	placebo
B_bi	B_bi	32.84 (−315.34, 380.05)	−145.49 (−547.44, 256.89)	48.85 (−248.73, 358.34)	85.53 (−255.15, 423.27)	71.02 (−297.99, 437.65)	51.95 (−194.91, 297.36)
BbM_16	−32.84 (−380.05, 315.34)	BbM_16	−177.77 (−576.53, 223.12)	15.82 (−280.99, 326.64)	52.64 (−287.25, 391.34)	38.1 (−331.14, 406.09)	19.24 (−226.41, 265.16)
LB	145.49 (−256.89, 547.44)	177.77 (−223.12, 576.53)	LB	196.68 (−164.38, 561.51)	230.45 (−164.1, 622.64)	215.71 (−208.59, 636.67)	197.25 (−122.72, 515.98)
LGG	−48.85 (−358.34, 248.73)	−15.82 (−326.64, 280.99)	−196.68 (−561.51, 164.38)	LGG	35.59 (−204.81, 264.86)	20.06 (−312.78, 344.63)	2.65 (−180.26, 174.66)
Lrh	−85.53 (−423.27, 255.15)	−52.64 (−391.34, 287.25)	−230.45 (−622.64, 164.1)	−35.59 (−264.86, 204.81)	Lrh	−14.26 (−374.69, 347.23)	−33.63 (−265.74, 201.09)
mix1	−71.02 (−437.65, 297.99)	−38.1 (−406.09, 331.14)	−215.71 (−636.67, 208.59)	−20.06 (−344.63, 312.78)	14.26 (−347.23, 374.69)	mix1	−18.5 (−295.93, 258.14)
placebo	−51.95 (−297.36, 194.91)	−19.24 (−265.16, 226.41)	−197.25 (−515.98, 122.72)	−2.65 (−174.66, 180.26)	33.63 (−201.09, 265.74)	18.5 (−258.14, 295.93)	placebo

### 3.7 Cluster analysis

**Figure 6:**
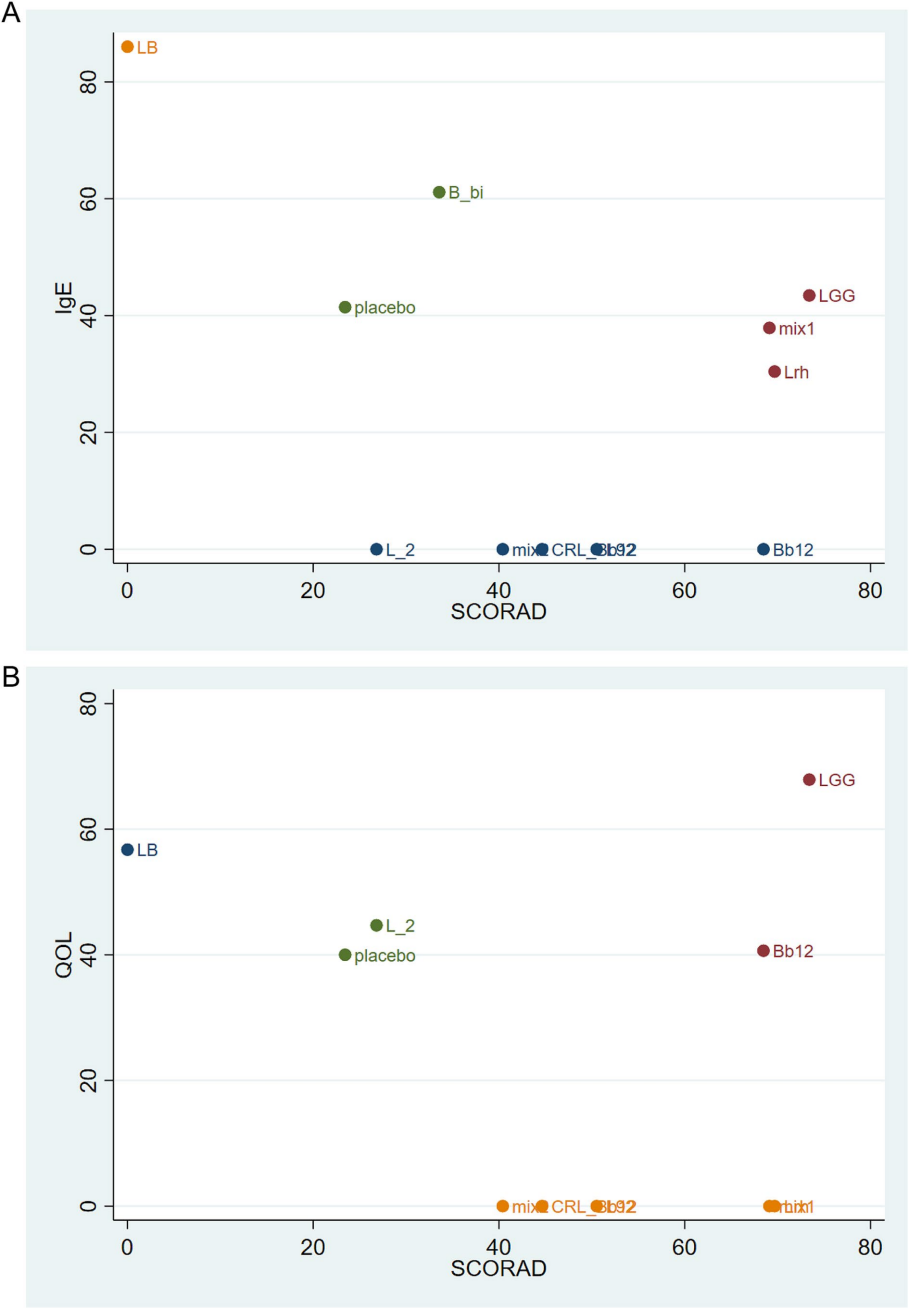
Cluster analysis based on SUCRA rankings. **(A)** Cluster analysis of SCORAD and IgE outcomes. **(B)** Cluster analysis of SCORAD and quality of life outcomes. Each point represents a probiotic intervention. The X and Y axes represent SUCRA values for each outcome. Interventions located in the upper-right quadrant exhibit high efficacy in both outcomes. Colors indicate grouping based on hierarchical clustering: interventions with similar efficacy profiles are grouped together. LGG and placebo cluster closely in both panels, indicating high and consistent efficacy.

Cluster analysis was conducted for SCORAD, IgE, and quality-of-life outcomes based on SUCRA rankings (Figures 6A,B). The results indicated that LGG and placebo were the most effective interventions. Figure 6A displays the SUCRA-based clustering of SCORAD and IgE scores, while Figure 6B visualizes clustering between SCORAD and quality-of-life outcomes. Each point in the plots represents an intervention, and the axes indicate the respective SUCRA values for each outcome. Interventions located in the upper-right quadrant show greater efficacy across both outcomes. LGG and placebo appear clustered in this quadrant, suggesting that both interventions exhibit robust and broad-spectrum effectiveness. The color of each point represents its group membership determined through hierarchical clustering, which helps identify interventions with similar outcome profiles.

### 3.8 Publication bias

**Figure 7:**
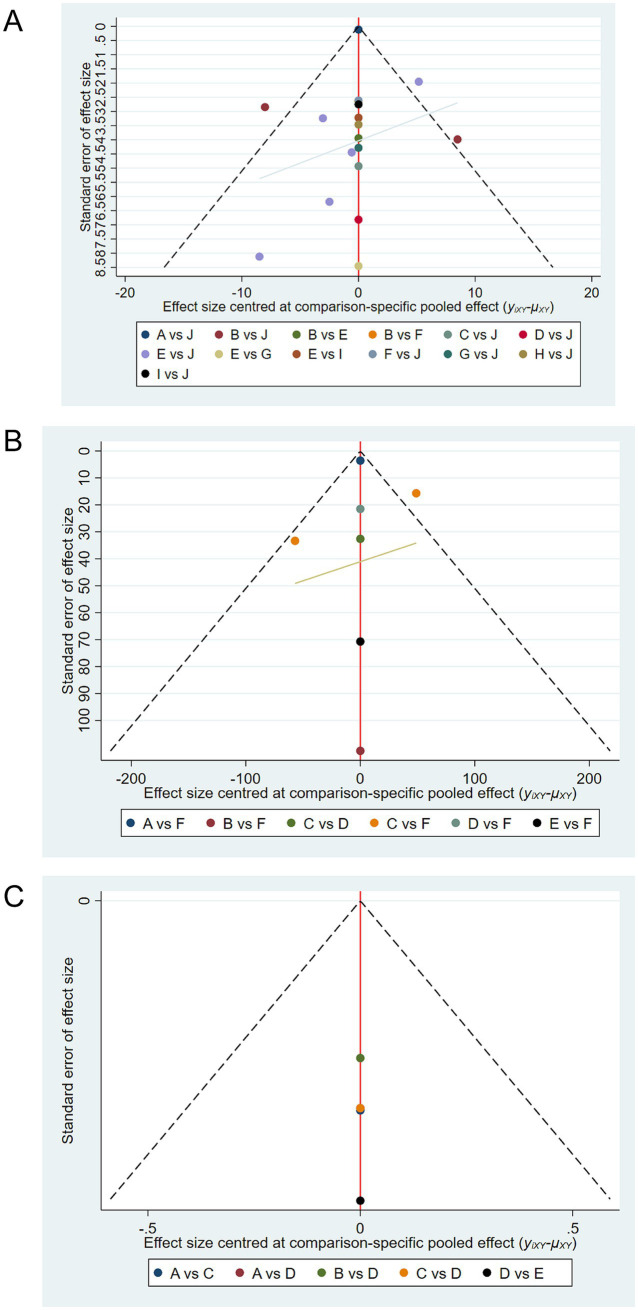
Comparison-adjusted funnel plots for publication bias. **(A)** Funnel plot for SCORAD outcomes. **(B)** Funnel plot for IgE outcomes. **(C)** Funnel plot for quality of life outcomes. Symmetry around the center line suggests absence of publication bias, while asymmetry suggests potential bias. Funnel plot for quality of life **(C)** exhibits mild asymmetry, indicating possible publication bias due to fewer included studies.

Comparison-adjusted funnel plots were drawn for SCORAD, IgE, and quality-of-life outcomes (Figure 7). Figure 7A shows the funnel plot for SCORAD outcomes, where studies are symmetrically distributed around the center line, suggesting low likelihood of publication bias. Figure 7B similarly shows a symmetrical distribution for IgE outcomes. However, Figure 7C presents a slight asymmetry for quality-of-life outcomes, indicating a potential publication bias, likely due to the limited number of included studies for this endpoint.

## 4 Discussion

This systematic review and network meta-analysis demonstrates the significant potential of probiotics as a complementary intervention for managing pediatric food allergies. Among the probiotics analyzed, *Lactobacillus rhamnosus GG* (LGG) emerged as the most effective strain for improving SCORAD scores and quality of life, while *Lactobacillus acidophilus* LB was most effective in reducing IgE levels. These results underscore the importance of probiotics in mitigating allergic inflammation, alleviating clinical symptoms, and enhancing overall well-being in children with food allergies.

A notable observation is the strain-specific efficacy of probiotics, which highlights the diverse immunomodulatory capacities of different microbial strains. While LGG consistently outperformed other probiotics across multiple clinical outcomes, mixed probiotics demonstrated variable results, possibly due to strain interactions that modulate their effectiveness. The heterogeneity among probiotic strains calls for careful consideration when designing future clinical interventions, with a focus on selecting strains that target specific immunological pathways relevant to food allergy pathogenesis.

The findings align with earlier research emphasizing the benefits of probiotics in allergic disease management. Prakoeswa et al. (41) similarly reported reductions in SCORAD and IgE levels with probiotic supplementation. They found that the supplement of *Lactobacillus plantarum* IS-10506 reduced SCORAD in children with atopic dermatitis. However, our study builds upon prior meta-analyses by incorporating quality-of-life metrics, which offer a holistic assessment of the burden of food allergies on both children and their caregivers. Unlike prior studies, which predominantly employed pairwise meta-analyses, this network meta-analysis provides a robust framework for comparing the relative efficacy of individual probiotic strains, even in the absence of direct head-to-head trials.

Despite some consistencies, our findings reveal notable differences compared to earlier studies. Previous meta-analyses often reported inconsistent results for probiotics other than LGG. These variations may stem from differences in study protocols, patient demographics, or probiotic formulations. By incorporating multiple clinical endpoints, our study provides a broader perspective. It suggests that strain-specific effects depend not only on the properties of the probiotic but also on the severity and type of food allergies being treated.

The strain-specific differences observed in this study highlight the complexity of using probiotics for food allergy management. LGG shows consistent efficacy, likely due to its ability to influence multiple immune and microbiota-related pathways (42, 43). By enhancing intestinal barrier function through the upregulation of tight junction proteins, LGG reduces allergen translocation and systemic immune activation (44). LGG also promotes the differentiation of regulatory T cells (Tregs) and decreases the activity of T-helper 2 (Th2) cells, which are central to allergic inflammation (45). These are the pathways through which probiotics exert their wideranging effects on SCORAD scores, IgE concentrations, and quality of life.

On the contrary, *Lactobacillus acidophilus* LB has stronger effects on decreasing IgE levels and may act through immunoglobulin switching regulation by the up-regulation of IL-10 production. These results suggest there are diverse mechanisms of action in different strains of probiotics that target the various steps in the allergic inflammation process. The option could give rise to tailor-made targeted therapies. The mixed probiotics produced mixed results in our study; this inconsistency may be due to antagonistic or nonsynergistic interactions among the strains. This emphasizes the need to select probiotic strains carefully when formulating multi-strain products to minimize the reduction of therapeutic efficacy.

Probiotics exert their therapeutic effects by regulating gut microbiota composition and functionality, enhancing intestinal barrier integrity, and modulating immune responses (46, 47). Specifically, beneficial strains like Lactobacillus and Bifidobacterium produce short-chain fatty acids (SCFAs), especially butyrate, which help maintain tight junction proteins and suppress mucosal inflammation. SCFAs also promote the differentiation of regulatory T cells (Tregs), leading to the downregulation of pro-inflammatory cytokines such as IL-6 and TNF-α, thereby shifting the Th1/Th2 balance towards immune tolerance (14, 15).

In addition, probiotics directly act on the GALT that regulate α4β7 + dendritic cells affecting antigen presentation. The interaction promotes immune tolerance by inhibiting Th2-mediated responses and increasing the production of anti-inflammatory cytokines such as IL-10 and TGF-β. Such immunological effects do not merely act to diminish allergic inflammation but also decrease IgE production, which is a key contributor to the symptoms of food allergy. The capacity of probiotics to act at various levels of the gut-immune axis emphasizes their future perspective of being a multi-targeting therapy.

In this meta-analysis, subgroup analyses displayed significant differences in probiotics’ effectiveness based on the type of allergy. Notably, children diagnosed with cow’s milk allergy (CMA) responded more favorably to probiotic interventions compared to those with peanut or mixed-food allergies. This observation is consistent with prior studies showing enhanced efficacy of *Lactobacillus rhamnosus* GG in managing CMA-related symptoms (48) In contrast, the therapeutic effects of probiotics appear more variable or limited in other allergen types, such as peanut allergy (38). A possible explanation for this discrepancy may lie in the underlying immunopathology associated with different allergens. For example, CMA is often associated with early-life dysbiosis and gut barrier dysfunction, which probiotics may more directly target. Conversely, peanut allergy often involves systemic immune sensitization that might be less responsive to microbiota-based modulation. These findings underscore the importance of considering allergen-specific pathophysiology when evaluating probiotic efficacy in pediatric populations.

These findings suggest that probiotics may be more effective in the early stages of allergic disease. Kukkonen et al. conducted a randomized, double-blind, placebo-controlled trial demonstrating that probiotic supplementation during pregnancy and early infancy significantly reduced the incidence of allergic diseases in children (49). More recently, Zakiudin et al. found that maternal probiotic supplementation was associated with a decreased risk of atopic dermatitis in offspring, potentially through modulation of inflammatory protein expression (50). These findings suggest that the timing of probiotic administration is critical, with early interventions offering greater benefits in allergy prevention. One can acknowledge several limitations of the study, in all fairness. The heterogeneity as was high across studies creates interpretation problems for studying the pooled results (51, 52). This heterogeneity underscores the urgent need for standardized protocols in probiotic research to enhance comparability across studies.

Another limitation is the reliance on published studies, which introduces the potential for publication bias. Negative or null results may be underreported, leading to an overestimation of probiotic efficacy. Additionally, the short follow-up periods in most studies limit our ability to assess the long-term sustainability of probiotic effects. Given that immune tolerance development is a gradual process, longer-term studies are essential to determine whether probiotics can induce durable improvements in allergic outcomes. Finally, inconsistencies in outcome reporting, particularly for quality-of-life measures, highlight the need for greater uniformity in study methodologies. Standardized reporting of endpoints, including SCORAD, IgE, and quality-of-life metrics, is critical for advancing the field and ensuring that future meta-analyses can build upon a robust evidence base.

The findings of this meta-analysis have important implications for clinical practice. LGG stands out as a highly effective strain for pediatric food allergy management, particularly for improving quality of life and alleviating allergic symptoms. Its consistent efficacy across these different endpoints suggests its integration into treatment protocols as a supportive therapy. *Lactobacillus acidophilus* LB, with this exquisitely marked effect on IgE reduction, is also another good candidate for effective immune modulation.

The variability in responses among strains and patient populations warrants a personalized approach to probiotic therapy. The type of allergy, bacterial profile, and severity of the disease should guide strain selection and dosing strategies. There should also be standardization in the various methods of probiotic regimens one applies, e.g., doses and duration of treatment, if these preparations are to yield maximal efficacy and reproducibility in clinical settings.

Future studies need to focus on large-scale trials using standardized methodology with prolonged follow-up to determine if probiotics indeed induce efficacy and sustain that efficacy. Mechanistic studies and individualized approaches based on microbiota profiles will help optimize therapy for food allergies in children. These findings highlight the urgent need for longer-term, well-funded trials to validate probiotic efficacy in pediatric populations.

## 5 Conclusion

In conclusion, this meta-analysis has indicated the promise of probiotics, and especially LGG, in the management of food allergies in children. Although the result is encouraging, the considerable heterogeneity of the studies includes the lack of standardized protocols and requires further research. This study provides the basis for future studies aimed at enhancing the use of probiotics in a clinical setting and advancing microbiota-based therapies for allergic diseases.

## Data Availability

The original contributions presented in the study are included in the article/Supplementary material, further inquiries can be directed to the corresponding authors.
