# Supplementary material for: Lactobacillus GG and other probiotics in pediatric food allergy treatment: a network meta-analysis
Source: Front Nutr. 2025 Jun 3;12:1565436. doi: 10.3389/fnut.2025.1565436 (PMC12172551; doi:10.3389/fnut.2025.1565436)
Supplement: Supplementary file 4 [file Table_2.docx]

Search Name: cochrane 525

Last Saved: 06/03/2024 19:52:42

Comment:

ID Search

#1 MeSH descriptor: [Probiotics] explode all trees

#2 MeSH descriptor: [Lactobacillus] explode all trees

#3 MeSH descriptor: [Bifidobacterium] explode all trees

#4 MeSH descriptor: [Gram-Positive Bacteria] explode all trees

#5 (Probiotic):ti,ab,kw OR (Lactobacillus):ti,ab,kw OR (Actinomyceta*):ti,ab,kw OR (Gram Positive Bacteria):ti,ab,kw

#6 #1 OR #2 OR #3 OR #4 OR #5

#7 MeSH descriptor: [Food Hypersensitivity] explode all trees

#8 MeSH descriptor: [Hypersensitivity] explode all trees

#9 (Food Hypersensitivities):ti,ab,kw OR (Food Allerg*):ti,ab,kw OR (Hypersensitivities):ti,ab,kw OR (Allerg*):ti,ab,kw

#10 #7 OR #8 OR #9

#11 MeSH descriptor: [Child] explode all trees

#12 MeSH descriptor: [Adolescent] explode all trees

#13 MeSH descriptor: [Infant] explode all trees

#14 MeSH descriptor: [Infant, Newborn] explode all trees

#15 MeSH descriptor: [Premature Birth] explode all trees

#16 (Children):ti,ab,kw OR (Adolescen*):ti,ab,kw OR (Teen*):ti,ab,kw OR (Youth*):ti,ab,kw OR (Infants):ti,ab,kw

#17 (Newborn*):ti,ab,kw OR (Neonat*):ti,ab,kw OR (Premature Births):ti,ab,kw OR (Preterm Birth):ti,ab,kw

#18 #11 OR #12 OR #13 OR #14 OR #15 OR #16 OR #17

#19 #6 AND #10 AND #18

#20 MeSH descriptor: [Randomized Controlled Trial] explode all trees

#21 (random*):ti,ab,kw

#22 #20 OR #21

#23 #6 AND #10 AND #18 AND #22
